# 

**Published:** 1887-12

**Authors:** 


### Medical News and Miscellany.

MARRIED: November 29, 1887, in Caldwell, Texas, at the residence of the bride's mother, Dr. Thos. O. Maxwell, of Fiskville, Travis county, (near Austin) to Miss Florence Porter, of Caldwell, Burleson county.

This seems to be a good (or bad) season for young doctors—they are going off like—we had nearly said—"hot cakes." But the strange part of it is that most of our young doctors get their brides from a distance, while Austin is literally blooming with the fairest of the fair, and scores of them! We congratulate Dr. Tom. with all our heart, and wish him a long life of happiness; and we can, and do congratulate Miss Florence, for the doctor has been considered a "catch," the despair of the girls hereabout, and the envy of the gents. We'll venture, more sheep's eyes and sly glances have been wasted on the handsome young sawbones than on most fellows, but, it seems, fate decreed that he was to find his destiny in Burleson. The doctor has settled in his cosy country home in the rich Del Valle settlement, and gone to work with renewed euergy, building up his practice and extending his fame as a safe and conscientious practitioner of the great healing science. May many years of honor and usefulness be his.

---

MARRIED: In Austin. Texas, November 24, at the Tenth Street Methodist Church, by the Rev. Dr. Pinson, Dr. A. L. Cocke, of San Marcos, to Miss Emma Earnest, of Austin. No cards.

We acknowledge the conrtesy of a special invitation to be present at the marriage, and also at the ante-nuptial banquet given by Dr. Cocke at the Driskill at 4 p. m., same day. On that occasion the gallant doctor entertained at a sumptuous dinner, a party of ladies and gentlemen to the number of thirty, friends from San Marcos, who came up to "see him off," together with some of his medical friends, resident in Austin. His health was drank in many a bumper of choice wine, and many and sincere were the wishes for a long life of happiness to himself and the fair one of his choice. The JOURNAL's best wishes attend them on their journey through life.

MARRIED, in Quincy, Illinois, December 21, Dr. Frank B. King, of Burnett, Texas, to Miss Lizzie Winston, a sister of Col. J. M. Lewis. We extend congratulations to our friend and neighbor, Dr. King, who is a member of the Travis County Medical Society, and wish him abundant happiness.

---

MARRIED: The Times is pleased to record the marriage, on Wednesday, the 30th ult., of Dr. T. M. Stone to Mrs. Sally Norsworthy, at Jasper. Dr. Stone is an elegant gentleman in every sense of the term, and his innate refinement and goodness of heart find congenial symphony in the characteristic graces of his pretty and accomplished bride.—*Colmesneil Times.*

